# Diagnostic performance of a rapid immunochromatographic test for the simultaneous detection of antibodies to *Theileria equi* and *Babesia caballi* in horses and donkeys

**DOI:** 10.1186/s13071-024-06253-1

**Published:** 2024-03-28

**Authors:** Frans Jongejan, Cheng Du, Elias Papadopoulos, Valeria Blanda, Santina Di Bella, Vincenza Cannella, Annalisa Guercio, Domenico Vicari, Sharon Tirosh-Levy, Amir Steinman, Gad Baneth, Sanna van Keulen, Iris Hulsebos, Laura Berger, Xiaojun Wang

**Affiliations:** 1 Vectors and Vector-Borne Diseases Research Programme, Department of Veterinary Tropical Diseases, Faculty of Veterinary Science, University of Pretoria, Private Bag X04, Onderstepoort, 0110 Republic of South Africa; 2 State Key Laboratory for Animal Disease Control and Prevention, Harbin Veterinary Research Institute, Chinese Academy of Agricultural Sciences, Harbin, China; 3 Laboratory of Parasitology and Parasitic Diseases, School of Veterinary Medicine, Aristotle University of Thessaloniki, University Campus, Thessaloniki, Greece; 4 Istituto Zooprofilattico Sperimentale della Sicilia “A.Mirri”, Palermo, Sicily Italy; 5 Koret School of Veterinary Medicine, The Hebrew University of Jerusalem, 7610001 Rehovot, Israel; 6 TBD International BV, BioScience Center, Runderweg 6, 8219 PK Lelystad, The Netherlands

**Keywords:** *Theileria equi*, *Babesia caballi*, Equine piroplasmosis, Horses, Donkeys, Immunochromatographic test, Competitive ELISA

## Abstract

**Background:**

Equine piroplasmosis is caused by two tick-borne protozoan parasites, *Theileria equi* and *Babesia caballi,*, which are clinically relevant in susceptible horses, donkeys, and mules. Moreover, equine piroplasmosis significantly constrains international trading and equestrian events. Rapidly diagnosing both parasites in carrier animals is essential for implementing effective control measures. Here, a rapid immunochromatographic test for the simultaneous detection of antibodies to *T. equi* and *B. caballi* was evaluated using samples from horses and donkeys collected in Greece, Israel, and Italy. The results were compared with an improved competitive enzyme-linked immunosorbent assay (cELISA) for detecting antibodies to both parasites using the same panel of samples.

**Methods:**

Blood samples were collected from 255 horses and donkeys. The panel consisted of 129 horses sampled at four locations in northern Greece, 105 donkeys sampled at four locations in Sicily, and 21 horses sampled at two locations in Israel. The rapid test and the cELISA were performed according to the manufacturer’s instructions, and the results were subjected to a statistical analysis to determine the sensitivity and specificity of both tests and their association.

**Results:**

The immunochromatographic test provided a result within 15 min and can be performed in the field, detecting both pathogens simultaneously. The overall coincidence rate between the rapid test and the cELISA for detecting antibodies against *T. equi* was 93% and 92.9% for *B. caballi*. The rapid test’s sensitivity, specificity, positive predictive value (PPV), and negative predictive value (NPV) for *T. equi* were above 91.5%. Sixteen samples were positive for both parasites in the rapid test and eight in the cELISA. Either test had no significant association between *T. equi* and *B. caballi* detection. The detection rates of both parasites were significantly higher in Italy than in Greece or Israel and in donkeys than in horses. The agreement for *T. equi* between the results of both tests was high in Greece (93.8%) and Italy (95.2%) and moderate in Israel (76.2%). For *B. *caballi, the specificity and NPV of the rapid test were high (94.2% and 98.3%, respectively), although the sensitivity and PPV were moderate (69.2% and 39.1%, respectively) due to the small sample size. However, for *B. caballi*, the sensitivity was higher with the rapid test.

**Conclusions:**

The rapid test detected *T. equi* and *B. caballi* simultaneously in the field, potentially replacing laborious cELISA testing and is recommended for import/export purposes. The test can also be helpful for the differential diagnosis of clinical cases, since seropositivity may rule out equine piroplasmosis since it does not indicate current or active infection.

**Graphical Abstract:**

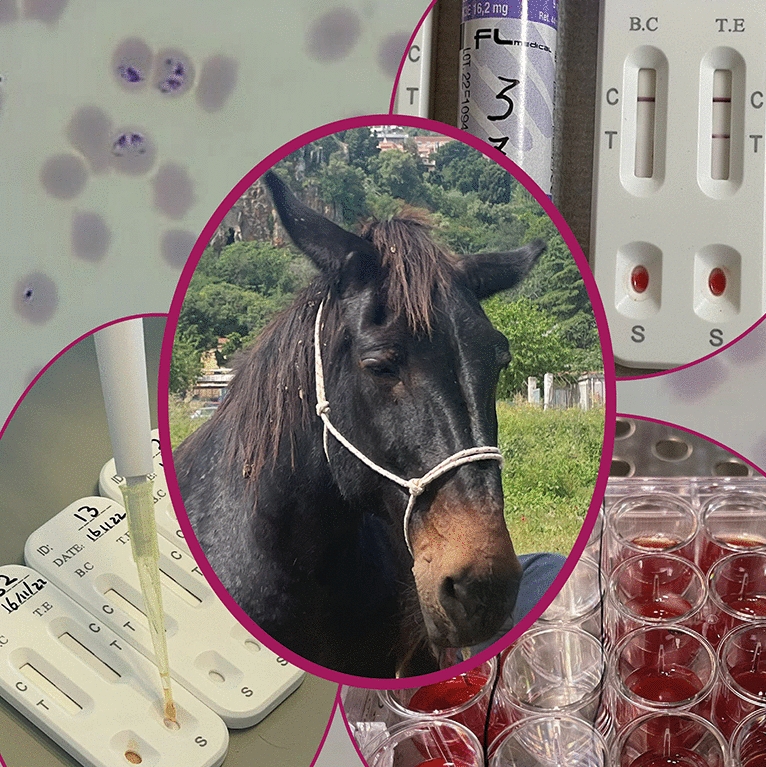

## Background

*Theileria equi* and *Babesia caballi*, causal agents of equine piroplasmosis, are distributed over a wide geographical area [1]. A broad range of tick species transmits both protozoan blood parasites [2]. Piroplasmosis is clinically relevant in susceptible horses, donkeys, and mules and is characterized by fever, hemolytic anemia, and clinical signs associated with progressive erythrocytic lysis. Although equine piroplasmosis is widely distributed in horses, donkeys, and mules, disease-free countries have introduced stringent animal movement restrictions to prevent the introduction of *T. equi* and *B. caballi* in carrier animals [1]. These regulations hamper international horse trading and constrain international equestrian events—for instance, the impact of a case of equine piroplasmosis in the Tokyo 2020 Olympic Games [3, 4]. Effective measures to control equine piroplasmosis in endemic countries depend on highly sensitive serological tests to detect animals carrying *T. equi*, *B. caballi*, or both parasites [1, 5]. A third species, *Theileria haneyi,* recently discovered in horses at the USA–Mexico border, further complicates the surveillance of equine piroplasmosis [6]. Although *T. haneyi* is less virulent than *T. equi* in horses, this third blood parasite must be considered when developing and applying methods for serodiagnosing piroplasmosis in horses [7, 8].

Parasitological examination of Giemsa-stained blood smears is helpful in acute and early infections but is not sensitive enough to detect carrier animals. *Babesia caballi* is a large intraerythrocytic protozoan parasite usually found in single, paired pyriform and ring forms. *Theileria equi* also appears in erythrocytes but is much smaller and rather polymorphic, with a typical formation of Maltese crosses evidence of dividing into four daughter cells. Indirect diagnostic techniques, such as complement fixation technique (CFT), indirect immunofluorescence antibody test (IFAT), and various enzyme-linked immunosorbent assays (ELISA) have been developed to detect parasite-specific antibodies in equine sera [1].

Currently, competitive ELISA and IFAT are used by reference laboratories tailored for international certification [11], and both tests are recommended by the World Organization for Animal Health (WOAH) [9–11]. Although polymerase chain reaction (PCR)-based methods for detecting *T. equi* and *B. caballi* DNA are available and generally more sensitive than the traditional methods, these tests have not yet replaced serological assays for international certification and monitoring [11–14]. However, these methods are suited for a fully equipped laboratory rather than in the field.

Recently, a rapid card test containing two colloidal gold immunochromatographic strips was developed to detect *T. equi* and *B. caballi* antibodies in equine serum [15]. This test employs a double-antigen-sandwich immunoassay format, wherein recombinantly expressed proteins are used to capture parasite-specific antibodies. Recognizing the gold-labeled antigens with their corresponding antibodies results in a visible color reaction [15]. The distinctive advantage of colloidal gold particles is that they can be directly observed without staining. Similar rapid tests are available for rapid diagnostic purposes at a low cost.

The rapid test includes recombinant *T. equi* erythrocytic merozoite antigen 1 (EMA1), known to induce specific neutralizing antibodies in infected animals and is thus suitable for use in a diagnostic assay [16]. Moreover, a 48 kDa merozoite rhoptry protein (BC48) has been previously identified as an appropriate diagnostic antigen for serologically detecting antibodies to *B. caballi* [16]. The test consists of two strips containing recombinant EMA1 and BC48 to simultaneously detect and distinguish antibodies of both pathogens.

The specificity of the rapid immunochromatographic card test has been determined previously by demonstrating that a broad range of bacterial, viral, and protozoan pathogens tested negative, and importantly, there was no cross-reaction between *T. equi* and *B. caballi* antibodies [15]. Furthermore, the test sensitivity was evaluated by comparing 476 serum samples from 15 provinces of China, with the results obtained from a commercial competitive ELISA kit marketed by VMRD (Pullman, WA). The coincidence rate of *T. equi* and *B. caballi* in the rapid test versus the cELISA was 96.4% (459 out of 476 samples) and 97.9% (466 out of 476 samples), respectively. Hence, the newly developed rapid card test agreed with the WOAH-recommended cELISA.

As a next step, a novel competitive ELISA was developed using recombinant proteins EMA1 and BC48, wherein the corresponding monoclonal antibodies were directly conjugated to horseradish peroxidase (HRP) [17]. The HRP-labeled monoclonal antibodies instead of enzyme-conjugated secondary antibodies significantly shortened the ELISA procedure for diagnosing antibodies against both parasites in equine blood samples. This novel cELISA protocol was also compared with the commercially available cELISA kit from VMRD (Pullman, WA). The coincidence rates between the novel ELISA and the commercial cELISA assays were based on 200 equine serum samples collected from horses in Inner Mongolia, 97.5% for *T. equi* and 98% for *B. caballi* [17].

This paper discusses the diagnostic performance of the rapid immunochromatographic test for the simultaneous detection of antibodies to *T. equi* and *B. caballi* in horses and donkeys. The advantages and potential use of a rapid test in the field eliminate sending samples to a lab and waiting for serological results for import/export purposes. A panel of blood and serum samples collected from horses and donkeys in Greece, Israel, and Italy was tested and statistically correlated with the results obtained with a novel competitive ELISA.

## Methods

### Sample collection

Blood samples for serum were collected from horses and donkeys at ten different farms. Between 4 and 48 animals were sampled on each farm. Inclusion criteria consisted of a predominantly outdoor lifestyle at ranches or farms exposed to ticks year-round. All animals were asymptomatic for piroplasmosis at the time of sampling. Blood samples were collected by venipuncture of the jugular vein of each animal into a sterile tube. Samples were drawn and examined in the rapid test by attending veterinarians in the field in Greece and Italy and subsequently sent to the lab for testing with the cELISA. Samples collected in Israel were directly shipped to the lab, where they were examined with both tests. Blood samples were drawn without a coagulant and were the source of a small amount of serum sufficient to be tested. Alternatively, ethylenediamine tetraacetic acid (EDTA) blood samples were also used, although their higher viscosity sometimes hampered the running of the sample inside the immunochromatographical test area.

### Colloidal gold immunochromatographic test

The antibody detection card of *Theileria equi* and *Babesia caballi* was performed according to the manufacturer’s instructions (National Engineering Research Center of Veterinary Biologics Corp, Harbin, China). Approximately 10 μl serum or whole blood was put into each sample hole. Subsequently, two drops of diluent were added, after which the sample ran through the immunochromatographic strip for 15 min. A signal at the test (T) line indicates a positive diagnosis, provided the control (C) line was also visible. A signal at the C line without a signal at the test line indicated a negative diagnosis of either *T.equi* or *B.caballi* [15].

### Competitive ELISA

*Theileria equi* and *Babesia caballi* cELISA antibody test kits were obtained from the National Engineering Research Center of Veterinary Biologics Corp, Harbin, China and used according to the manufacturer’s instructions. Serum samples and internal positive and negative controls were diluted 1:1 in phosphate-buffered saline (PBS) (0.1 M and pH 7.4). Next, 100 µl of the serum samples and controls were added to the antigen-coated 96-well plate and then incubated for 30 min at 37 °C. After the incubation, plates were washed three times with 250 µl washing buffer (PBS with 0.1% Tween) per well. The liquid was shaken out, and the plate was tapped dry after each wash. After the third washing step, 100 µl of HRP-conjugated IgG was added to each well, and each plate was incubated for 30 min at 37 °C. After that, plates were washed as before, and subsequently, 100 µl of substrate solution was added to each well and incubated for 10 min at 37 °C in the dark. As a last step, 50 µl of 2 M H_2_SO_4_ was added to each well to stop the reaction. Plates were read on a portable microplate reader at an optical density of 450 mm (The Absorbance 96, Byonoy GmbH, Hamburg, Germany). The percentage inhibition equal to or greater than 40% identified an antibody-positive sample, and the sample was regarded as antibody-negative below < 40% [17].

### Statistical analysis

The sensitivity and specificity of the positive predictive value (PPV) and negative predictive value (NPV) of the rapid test were evaluated, considering the ELISA results as the gold standard for diagnosis. Interactions between parameters (animal species, farm, and country) and seropositivity by each method and the rate of agreement between both methods were evaluated using the chi-square test.

## Results

### Study population

**Fig. 1:**
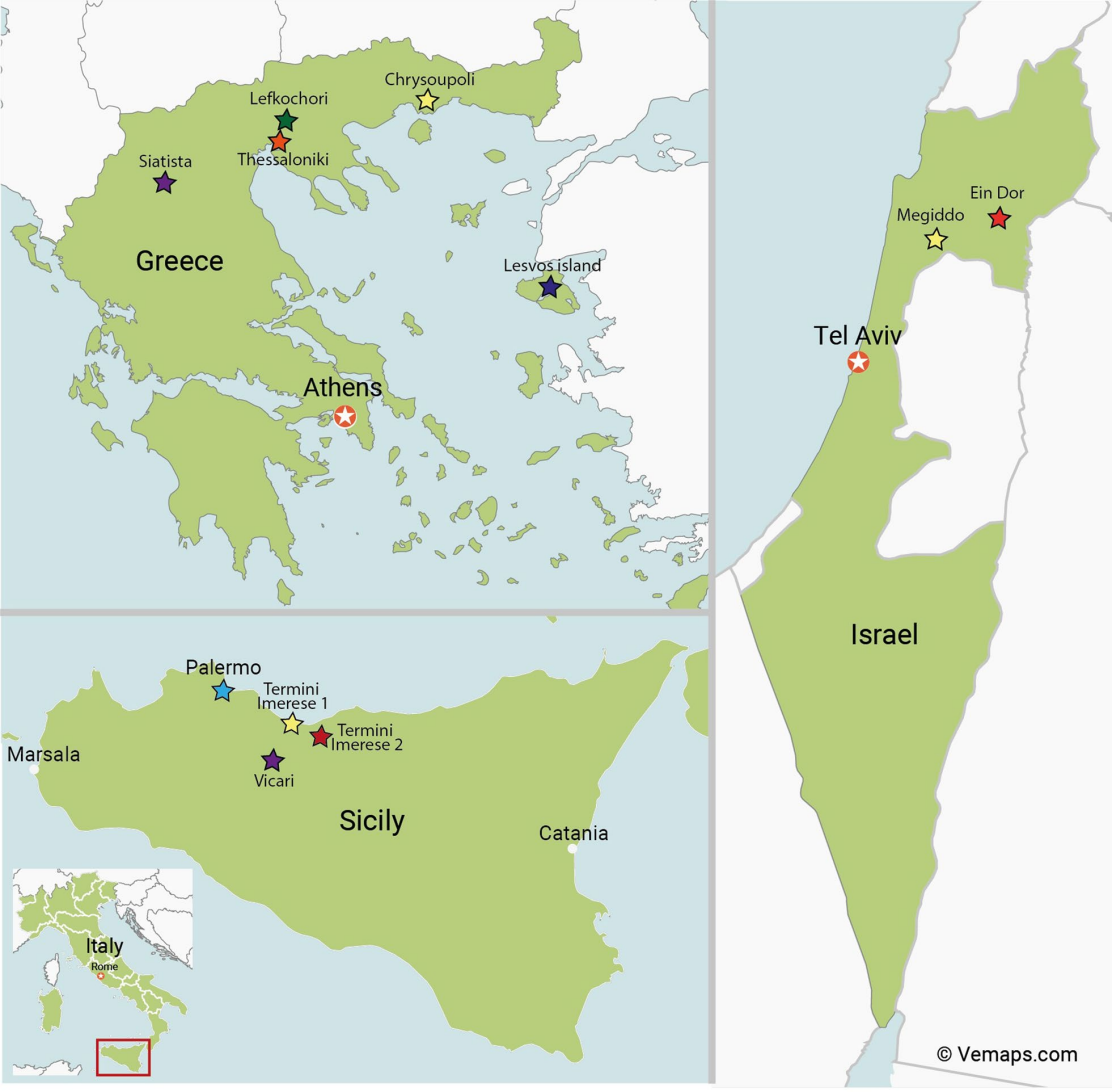
Geographical locations where horses and donkeys were sampled: four different locations in northern Greece (*n* = 129), four locations in Sicily, southern Italy (*n* = 105), and two locations in Israel (*n* = 21)

Blood samples for serum or samples drawn into EDTA were collected from 255 horses and donkeys. The panel consisted of 129 horses sampled at four locations in northern Greece, 105 donkeys at four locations in Sicily, and 21 horses at two locations in Israel (Fig. 1). Horses of approximately equal sex distribution sampled at four locations in northern Greece varied in age between one and 20 years, with a median of 9.9 years. Several animals were infested with ticks, which were not collected for further examination. In Italy, Ragusano donkeys, an autochthonous breed from the Mediterranean island of Sicily, where they attained special conservation status, were sampled at four farms and kept together with other livestock species. Several donkeys were infested with adult *Rhipicephalus bursa* ticks, a known vector of *B. caballi*. In Israel, horses were sampled at two locations where equine piroplasmosis had previously been identified.

### Serological detection of both parasites

Using the rapid test, 137 samples (53.7%) tested positive for *T. equi*, and 23 (9.0%) samples tested positive for *B. caballi* (Table 1). Sixteen of the samples were positive for both parasites. There was no significant association between *T. equi* and *B. caballi* detection in the rapid test (*P* = 0.110). Using the cELISA, 139 of the 255 samples (54.5%) tested positive for *T. equi*, and 13 (5.1%) tested positive for *B. caballi* (Table 1). Eight of the samples were positive for both parasites. There was no significant association between *T. equi* and *B. caballi* detection in the cELISA (*P* = 0.601).

**Table 1:** Comparative diagnostic performance of the rapid immunochromatographic test with the competitive ELISA for detecting antibodies to *Theileria equi* and *Babesia caballi*

		Rapid immunochromatographic test results: no. (%)					
		*T. equi*			*B. caballi*		
		Positive	Negative	Total	Positive	Negative	Total
cELISA test result: no. (%)	Positive	129 (50.6)	10 (3.9)	139 (54.5)	9 (3.5)	4 (1.6)	13 (5.1)
	Negative	8 (3.1)	108 (42.4)	116 (45.5)	14 (5.5)	228 (89.4)	242 (94.9)
	Total	137 (53.7)	118 (46.3)	255 (100)	23 (9.0)	232 (91.0)	255 (100)
% agreement				92.94%			92.94
Kappa				0.857			0.465
Sig (chi-square)				< 0.001			< 0.001
Sensitivity				92.8%			69.2%
Specificity				93.1%			94.2%
PPV				94.2%			39.1%
NPV				91.5%			98.3%

### *Theileria equi* serological detection

The overall coincidence rate between the rapid test and the cELISA for *T. equi* was 93% based on 129 positive and 108 negative samples out of 255 (Table 1). The null hypothesis of no association between the tests for *T. equi* was rejected (chi-square test = 312.64, *df* = 1, *P* < 0.001). The agreement between the results of both tests was almost perfect (*K* = 0.857). The sensitivity, specificity, PPV, and NPV of the rapid test were all above 91.5% (Table 1).

The detection rate of *T. equi* was significantly different in different farms, countries, and animal species, both by ELISA and the rapid test (all *P* < 0.001) (Table 2). The detection rates were significantly higher in Italy than in Greece or Israel and in donkeys than in horses (both *P* < 0.001); however, since all donkeys were sampled in Italy, these factors could not be evaluated independently. The agreement between the results of both tests was high in Greece (93.8%, *K* = 0.861, and *P* < 0.001) and Italy (95.2%, *K* = 0.848, and *P* < 0.001) and moderate in Israel (76.2%, *K* = 0.549, and *P* = 0.007). However, the agreement did not differ significantly between horses and donkeys (*P* = 231) (Table 2).

**Table 2:** Comparison of the rapid immunochromatographic test with the competitive ELISA for detecting antibodies to *Theileria equi* in horses and donkeys presented per country (Greece, Israel, and Italy)

		Rapid immunochromatographic test result for Theileria equi								
		Greece			Israel			Italy		
		Positive	Negative	Total	Positive	Negative	Total	Positive	Negative	Total
cELISA test result: no. (%)	Positive	39 (30.2)	8 (6.2)	47 (36.4)	8 (38.1)	0 (0)	8 (38.1)	82 (78.1)	2 (1.9)	84 (80)
	Negative	0 (0)	82 (63.6)	82 (63.6)	5 (23.8)	8 (38.1)	13 (61.9)	3 (2.9)	18 (17.1)	21 (20)
	Total	39 (30.2)	90 (69.8)	129 (100)	13 (61.9)	8 (38.1)	21 (100)	85 (81.0)	20 (19.0)	105 (100)
% agreement				93.79%			76.19%			95.24%
Kappa				0.861			0.549			0.848
Sig (Chi-square)				< 0.002			0.007			< 0.001

### *Babesia caballi* serological detection

The overall coincidence rate between both tests for *B.caballi* was 92.9% based on nine positive and 228 negative horses out of 255 (Table 1). A Chi-square test (*χ*^2^ = 29.8, *df* = 1, *P* < 0.001indicated a significant test association. The agreement between the results of both tests was moderate (*K* = 0.465). Although the specificity and NPV of the rapid test were high (94.2% and 98.3%, respectively), the sensitivity and PPV were moderate (69.2% and 39.1%, respectively). For *B. caballi*, the sensitivity was higher with the rapid test; 14 samples tested positive on the rapid test were negative with the cELISA. On the other hand, only four samples positive with the cELISA were negative for the rapid test (Table 1).

The detection rate of *B. caballi* was significantly different in different farms, countries, and animal species, both by ELISA and the rapid test (all *P* < 0.008) (Table 3). The detection rates were significantly higher in Italy than in Greece or Israel and in donkeys than in horses (both *P* < 0.001); however, since all donkeys were sampled in Italy, these factors could not be evaluated independently. The agreement between the results of both tests was moderate in Greece (97.7%, *K* = 0.562, *P* < 0.001) and Italy (86.7%, *K* = 0.427, *P* < 0.001) and could not be evaluated statistically for Israel, since none of the samples tested positive in the ELISA test (95.2% agreement). The agreement was also significantly higher in horses than donkeys (*P* = 0.001) (Table 3).

**Table 3:** Comparison of the rapid immunochromatographic test with the competitive ELISA for detecting antibodies to *Babesia equi* in horses and donkeys presented per country (Greece, Israel, and Italy)

		Rapid immunochromatographic test results for *Babesia caballi*								
		Greece			Israel			Italy		
		Positive	Negative	Total	Positive	Negative	Total	Positive	Negative	Total
cELISA test result: no. (%)	Positive	2 (1.6)	0 (0)	2 (1.6)	0 (0)	0 (0)	0 (0)	7 (6.7)	4 (3.8)	11 (10.5)
	Negative	3 (2.3)	124 (96.1)	127 (98.4)	1 (4.8)	20 (95.2)	21 (100)	10 (9.5)	84 (80)	94 (89.5)
	Total	5 (3.9)	124 (96.1)	129 (100)	1 (4.8)	20 (95.2)	21 (100)	17 (16.2)	88 (83.8)	105 (100)
% agreement				97.67%			95.23%			86.67%
Kappa				0.562			Na			0.427
Sig (chi square)				0.001			Na			< 0.001

## Discussion

A meta-analysis of the seroprevalence data in equids in European countries demonstrated an estimated prevalence of 30% and 8% and a prevalence of 25% and 2% for *T. equi* and *B. caballi*, respectively [18, 19]. This guided us to the Mediterranean basin in southern Europe in selecting suitable areas to identify carriers of equine piroplasmosis, whereby the expected prevalence was significantly higher than, for instance, in the Netherlands [20].

In Greece, ecological niche modeling has been utilized to predict the geographic range and to investigate clusters of infections for equine piroplasms [21]. Land cover, followed by temperature, was the most important environmental factor contributing to Greece’s ecological niche modeling. Significant clusters were detected for *T. equi* and *B. caballi* in Greece’s North and Central regions, respectively, with substantial equine populations. As a result, our survey focused on northern Greece. A serological previous study and genotyping of *Theileria* isolates provided a further basis for the efforts undertaken in Greece [22, 23] (Fig. 1).

In Israel, a previous study wherein the parasite loads of horses infected with *T. equi* or *B. caballi* were quantified to distinguish between infections resulting in disease and subclinical cases provided a basis for the current survey [24]. Moreover, the prevalence of *B. caballi* and *T. equi* has been studied in subclinical horses in Israel using serological methods and comparing them with molecular techniques [25, 27, 28].

Several studies have been conducted in Italy regarding the prevalence of *T. equi and B. caballi* and their clinical relevance in donkeys [26, 27]. This led us to Sicily, where the prevalence and pathogenicity of both parasites had been studied in protected populations of indigenous Ragusano donkeys (Fig. 1).

The rapid test was well received and used under field conditions in Greece and Italy. Blood samples were drawn without a coagulant and were the source of a small amount of serum sufficient to be tested. Alternatively, EDTA blood samples were also suitable, although their higher viscosity sometimes hampered the running of the sample inside the test area. More importantly, the sample volume required further precision when the plastic pipette was used, which was provided with the rapid test. The protocol stated to use 10 µl of a serum sample. However, one drop from the provided pipette was approximately 15 µl. This difference may affect the intensity of the visual test signal.

A further observation relates to the test’s reading time of 15 min. To illustrate this point, two strong positive and two low positive sera in the cELISA for *T. equi* or *B. caballi* were examined after 15 min and again after 30 min of the test. A distinct line was present in the two highly reactive samples within minutes, well before 15 min had passed. However, a faint line became visible in the two low positive samples after 30 min and did not increase further. It was also observed that specific signals never faded over time, which is convenient if one needs to reread the test result. Finally, when some samples were retested up to five times, results were consistent for both parasites, which adds to the confidence in the rapid test.

The sensitivity, specificity, PPV, and NPV of the rapid test were all above 91.5% (Table 1). The high values show that the test is accurate. Moreover, there was no significant association between *T. equi* and *B. caballi* detection in the rapid test (*P* = 0.110). This analysis shows that the tests are independent, and there is no cross-reactivity between both parasites. ELISA kits for equine piroplasmosis are available commercially but require multiple steps, including dilution of serum, incubation of primary antibody, and incubation of secondary antibody, as well as washing at each step. Therefore, a competitive ELISA with an improved protocol was developed by introducing a direct HRP conjugation [17]. The coincidence rates between this novel ELISA and the commercial cELISA assays were 97.5% for *T. equi* and 98% for *B. caballi.*

Moreover, the coincidence rate of *T. equi* and *B. caballi* in the rapid test versus the commercial cELISA, also previously determined, was 96.4% and 97.9% [15]. Hence, it was decided to compare the rapid test with the novel ELISA in this paper. It was found that the overall coincidence rate between the rapid test and the cELISA for *T. equi* was 93% based on 129 positive and 108 negative samples. The overall coincidence rate between both tests for *B. caballi* was 92.9% based on 9 positive and 228 negative horses (Table 1).

False-negative results have been reported with enzyme-linked immunosorbent assays based on RAP1 and EMA recombinant antigens for the serodiagnosis of equine piroplasmosis. For instance, the commercial cELISA did not reveal any positives for *B.caballi* in carrier horses in Israel, which, however, were clearly positive in the indirect immunofluorescence test (IFAT) [24]. For *B. caballi*, these limitations have been attributed to the high genetic diversity of RAP1 proteins among different isolates [28]. We need to confirm that the antigen used in the rapid test is more sensitive than the supposed gold standard ELISA [29]. It is expected that neither the EMA1-based rapid test nor cELISA will detect *T. haneyi* due to the absence of EMA1 in *T. haneyi*. For this purpose, a specific ELISA based on another merozoite antigen has been developed to detect equine antibodies to *T. haneyi* [30]. It remains to be demonstrated to what extent the rapid test can be improved to compensate for these shortcomings, whereby a larger sample size is required, particularly from Israel, where the rapid test could also have a small percentage of false negatives.

Although IFAT and cELISA remain the primary choices as regulatory tests in the international horse trade, the rapid test is recommended for inclusion into the WOAH guidelines for diagnosing equine piroplasmosis with a pen-side test. False negative test results have negative consequences, which could be avoided by using the rapid test before competing in endurance or other equestrian events.

## Conclusions

Our data support the introduction of rapid tests into the international arena to monitor and control equine piroplasmosis. Rapid on-site detection of antibodies to *T.equi* and *B.caballi* brings immediate value to horse owners since horses may be refused to participate in global events and impact trade value when tested positive. Finally, the rapid test will also be helpful in addition to PCR in equine clinics to rule out piroplasmosis in a differential diagnosis, since seropositivity does not necessarily imply current or active infection.

## Data Availability

TBD International BV, situated in Lelystad in the Netherlands, distributes the rapid test for equine piroplasmosis under the tradename PiroDuo^®^.

## Abbreviations

EMA: Erythrocytic merozoite antigen
RAP: Rhoptry antigen protein
HRP: Horseradish peroxidase
